# Richmond Academy of Medicine and Surgery

**Published:** 1897-03

**Authors:** M. W. Peyser

**Affiliations:** Secretary; Richmond, Va.


### *SOCIETY REPORTS.*

---

### RICHMOND ACADEMY OF MEDICINE AND SURGERY.

RICHMOND, VA., January 26, 1897.

Dr. J. N. Upshur, President, in the chair.

Dr. J. A. Hodges read a paper on "Tubercular Meningitis."

From whatever standpoint viewed, tubercular meningitis is a most interesting disease.

The consideration of its bacillary origin, its peculiar mode of invasion, its stubborn resistance to all known therapeutic measures, its appalling record of mortality, being the most directly fatal of any disease of early childhood; either or all are questions inviting the interest and enthusiasm of every student of disease.

It may almost be called an incurable disease; for, notwithstanding three cases of its happy termination are reported, it is not at all improbable that an incorrect diagnosis was a source of error even with those distinguished clinicians.

Discarding for the present other interesting and instructive features, I desire especially to direct attention to its *diagnosis*, for I consider this of paramount importance, in not so much that its fatal termination can be avoided, as it is at present treated, but that the ends of scientific exactness in this as in other diseases may be attained, and that future errors of prognosis may be avoided.

I am persuaded from personal practice and from my experience as consultant, that many cases of this disease have been incorrectly interpreted by the attending physician, and the significance of many of its varied symptoms overlooked, until its unfortunate termination revealed the correct nature of the illness. A melancholy experience with two such cases is a lesson still indelibly impressed upon my mind, and it was this that first attracted my attention to my lamentable error in diagnosis; for, deceived by the mild train of symptoms before coma supervened, I overlooked the danger that was impending, and gave an encouraging prognosis! When the symptoms that shall now be detailed are considered, is it possible that others may realize that they too may have to plead guilty to such a charge of mistaken diagnosis?

Tubercular meningitis presents a preponderance of nervous symptoms, and is a distinctively bacillary disease, being peculiar to childhood, and usually attacking children between two and ten years of age. The membranes involved are the same as those in simple meningitis, but the anatomical lesions differ, for the latter affects the pia and arachnoid on the convexity of the brain, while a meningitis of tubercular character attacks those membranes at the base.

The prodromal symptoms are vague, and for this reason are apt to be misleading. As much as any clinician, I abhor the division of any disease into arbitrary stages, being well aware how variable are the manifestations, but for the sake of clearness I prefer to so divide the symptoms of the disease; for, as a rule, there is a regular sequence in them; and it is only from a contemplation of this succession of symptoms, when associated with other manifestations, that the causes of the ominous changes which occur in the later periods is suspected and can be diagnosed.

I believe that too great importance cannot be attached to the mode of invasion of this disease as a diagnostic point. In simple meningitis there is a stormy onset, and a convulsion ushers in the scene, while in tubercular meningitis the convulsion is usually one of the last acts in the sad drama. In tubercular meningitis this period of invasion is so peculiar that it cannot be mistaken by the careful observer, if the facts which are of such import in the determination of this particular disease are faithfully elicited.

The initiatory symptoms are so indefinite as often to be deceptive. The child is not at once seized with a sudden illness. There is simply a *gradual failing of the general health,* and the only physical sign of disease is a more or less evident emaciation. This change in the condition of the child also is a variable one, being rapid and striking in some while in others it is scarcely noticeable. In all cases, however, the emaciation becomes at last a marked feature of the case.

During the progress of the change another symptom, which may be termed a *moral change*, develops itself, since it is manifested in both the *habits and temper* of the child; for the little patient, according to its individual temperament, may exhibit all the phases and variations of its nature. Formerly bright and playful, it now becomes morose and sluggish, or irritable and disagreeable. This change of disposition is often supposed to be a reflex condition, due to some primary cause of disease in childhood, such as dentition or some digestive disturbance; but if carefully investigated, no such causative factor is usually discovered.

This moral change in the usual life of the child is not an evanescent one, but is continuously and progressively more marked; and its development should convey a deeper significance to the experienced practitioner.

*First Stage.*—Generally coincident with the moral change another symptom supervenes which is significant, namely, the existence of a *headache.* This cephalalgia in these cases is characteristic, for it is violent, paroxysmal and generally localized, as evidenced by the actions of the little sufferer. *Vomiting* is usually associated with it, and it is not the ordinary vomiting from gastric disturbances, but the true cerebral vomiting, propulsive or regurgitative in character, unaccompanied by pain or nausea, as for instance in the vomiting observed in hepatic or abdominal disorders. These symptoms, accompanied by a mild degree of pyrexia, the temperature most usually exceeding $100\frac{1}{2}^{\circ}$ F., a slightly accelerated pulse, wakefulness, photophobia, constipation, contraction of the pupils, with possibly some hyperesthesia of the abdomen, constitute what I would denominate the first stage. In proportion to the intensity of this disease in individual cases will the tardy or rapid succession of the symptoms noted above be manifested, for, generally, in affections of the brain, symptoms of irritation and excitation appear first; but sometimes the symptoms of profound depression are initiatory, and this regular succession of phenomena is not so prominent. These prodromal symptoms of the first stage may extend over a period ranging from one to several weeks.

*Second Stage.*—The second stage of the disease (always supposing there is a regular succession of phenomena) ushers in more pronounced symptoms, and there appears to be an increase in their intensity. The temperature rises slightly, and a peculiar *embarrassment of respiration* is noticeable, the patient sighing deeply and long. The pulse now becomes remarkably slow, and there is generally an interference in its rhythm which corresponds to the change in the respiration. The eyes half close, and the eyeballs move slowly in a lateral direction, one or both pupils dilating. At this period there are sudden flushes of the face, and a kind of mottling appearance upon the skin, over which, if the finger is drawn, red marks are left temporarily; and a sunken, retracted condition of the abdomen is evident. While these two conditions are characteristic in this affection, they are not pathognomonic. During this stage there is also developed a *cephalic cry*, which once heard is never forgotten. The *remission in the fever*, which is apt to occur now, is often deceptive and may lead to false hopes. *Increased somnolence* becomes apparent towards the close of this stage, and in reality is but the beginning of the end.

*Third Stage.*—The foregoing train of symptoms is but introductory to the last stage, where *coma* is the prominent feature, overshadowing all other conditions. Evidences of vital depression and of organic implication are now more marked, and slight twitchings around the mouth and eyelids, or a convulsion, followed by ptosis-strabismus, or paralysis of the face or extremities, promptly follow. The head may become retracted, and all the other symptoms increase in severity till the fatal termination ensues, after a period of three or four weeks.

Not all cases pursue this definite course, for in some children the symptoms may be much milder, while in others there may be so much somnolence from the beginning as to obscure many of them. In fact, to say anything positive about the course of this disease is impossible; it is sometimes acute, sometimes chronic, sometimes presenting long intermissions, and sometimes steadily progressive. Even the manner of onset may vary much, and the different stages or periods so coalesce as to be scarcely distinguishable from each other.

*Differential Diagnosis.*—From the foregoing, it is evident that an absolutely positive diagnosis of tubercular meningitis can be made only when a full and true history of the successive phenomena of the illness can be obtained; or when the symptoms of a tubercular meningitis supervene upon tubercular lesions that have previously existed in other organs. Some of the diseases with which tubercular meningitis may be confounded are:

1. Simple meningitis. In this disease, the prodromal symptoms are wanting; the history of tuberculous infection or predisposition is lacking; the onset is more sudden, and the course is more quickly run.

2. Cerebro-spinal meningitis. When this disease occurs, endemically or sporadically, the vaso-motor disturbances occur earlier, and the characteristic eruption is apt to be present.

3. The hydrocephaloid state. This condition in many particulars is so similar to a tubercular meningitis that it can be differentiated only by a history of some gastro-enteric disturbance prior to the supervention of the brain symptoms.

4. Malarial fever. The history of malarial infection, or the proper administration of quinine, usually solves the question of diagnosis; otherwise, an examination of the blood must be made for the specific germ.

5. Typhoid fever. In this disease, the symptoms of a cerebral character very closely simulate a tubercular meningitis, especially if the temperature curve be atypical, as is frequently the case. A microscopical examination of the blood is often necessary as a crucial test, but, clinically, it is always well to remember that in typhoid there is no peculiar or rhythmical disturbance of the respiration, as in meningitis from a tuberculous infection.

*Treatment.*—When the prognosis of this disease can be summed up in one word, death, it is evident that so far no treatment has ever been successful. The multiplicity of remedies suggested corresponds only with their inefficiency.

Recognizing personally the utter helplessness of all known measures of therapeusis, as employed in the past, to combat this fatal malady, I purpose in the future to use *anti-tubercle serum*, or if that proves inefficient, to employ *surgical means* to remove the intra-cranial pressure. I am aware that such a procedure would be radicalism in the extreme, but it is a principle which is applied to all other forms of internal pressure and engorgement, and the necessities of such cases demand the application of any measures based on scientific warrant. The ventricles of the brain have been drained and flushed with antiseptic solutions in other diseases, then why not in this? Surely, the phenomenal successes secured in some cases by simply flushing the peritoneum for tubercular disease ought to be encouraging and stimulating to like endeavors in these cases.

In the treatment of this disease, the past is a history of successive and multiplied failures as regard the use of medicines. Is it a vain hope, then, that in the future surgery may solve the mystery of its successful treatment?

DISCUSSION.

Dr. Wm. S. Gordon said Dr. Hodges had conferred a favor upon the Academy by calling attention to the disease under discussion. He could look back to his cases and bear testimony to the fact that errors in diagnosis were easily made; and that we were frequently liable, in the early stages of tubercular meningitis, to mistake it for typhoid fever or some other affection.

Dr. Hodges had referred to the gradual onset and late convulsions, as differential diagnostic points; but it was well to bear in mind that the malady was sometimes ushered in with a convulsion, the prodromata being absent or hardly noticeable. One such typical case he remembered seeing some years ago. Another point worthy of mention was the vacillating pupil in the early stages. The pupil was moderately dilated, contracted promptly but incompletely to a bright light, but returned rapidly to the previous dilatation in the presence of the light. Wide and fixed dilatation, with respiratory embarrassment, was observed in the late stages, and was due to pressure upon the bulb centers by effusion. In connection with the differential diagnosis, he would like to ask Dr. Hodges whether he believed in simple, so-called, idiopathic meningitis, irrespective of traumatism?

Dr. Hugh M. Taylor had recently treated three cases. He knew of no more appalling condition. In his own and in the experience of others it had been attended with a mortality of one hundred per cent. If a case diagnosed as tubercular meningitis got well by bromides, calomel and the iodides, he should doubt the correctness of his diagnosis. The cases referred to were characterized by sudden invasion, rapid progress, and a clear history of infection in each. One was a strong, robust little boy, age five years; he was a picture of vigorous childhood. His father was in the last stages of pulmonary tuberculosis. The mother was urged not to sleep in the father's room and not to let the child do so; and was told of the contagiousness of the disease. This advice was not respected. With no premonitory symptoms, cerebral irritation, cerebral effusion, cerebral pressure, stupor, convulsions, coma, and death quickly ensued. From vigorous health to death occupied only eight days.

A second case occurred in a child, a victim of quiescent morbus coxarius. Four or five days before his death this child was on the street as well as he had been for years. Cerebral irritation, quickly followed by cerebral effusion, stupor, coma, convulsions, and death ensued.

The third case was that of a child, aged three years, with active spondylitis in the upper dorsal region. Pachymeningitis of the spinal dura ushered in an infection with rapid extension to the brain envelopes, with effusion, pressure effects, stupor, coma, and death in a few days.

As he watched these cases with a full knowledge that there was but one possible outcome—speedy death—he could but agree with others that some other plan of treatment than that usually followed was justifiable. A mortality of one hundred per cent. could not be made worse; and force the conviction that such a desperate condition warrants heroic remedial resources. Diffused or circumscribed septic pleuritis, pericarditis, and peritonitis and joints, called for operative interference. In the brain, apart from the septic processes, intra-cranial pressure incident thereto must be quickly removed. Mixed infection, he thought, must play an important role in the intra-cranial sepsis, the tubercular deposits and focus acting as factors which diminish tissue resistance and favor mixed infection. Removal of the effects of intra-cranial infection and its consequences—brain disorganization and perturbed function—could only be accomplished by operative interference. We do not hesitate to drain other serous cavities, and irrigate them, and we should not, in the face of such dire necessity, fail to do something, hesitate to open, drain, and irrigate when we have the ventricles distended with septic fluid in meningeal inflammation. In 1873, Dr. S. W. Gross urged the value of puncture, irrigation, and drainage, and his tabulated reports at that time showed 45 per cent. of recovery. If such marked success could be obtained twenty-five years ago, with improved technique and asespsis we should hope for a decided lessening of the death by operative interference. Etiologically, he thought we should regard the intra-cranial pressure occurring in cases of tubercular meningitis as mixed infection; and, clinically, should be placed in meningitis, with streptococcus infection from any other cause. Tubercular peritonitis had come within the pale of rational surgery, and it was along the lines of ventricle or spinal puncture and drainage and irrigation that we must look for advance in the treatment of tubercular meningitis and its sequences, effusion, intra-cranial pressure, stupor, coma, convulsions, and death.

Dr. Gordon, referring to Dr. Taylor's remarks, said it had occurred to him during the discussion that temporary abatement of the symptoms might be obtained by tapping and aspirating the spinal membranes in the lumbar region, but he did not know that the procedure had been conceived or accomplished. Whether this operation would do for the disease what drainage of the abdomen had done for tubercular peritonitis was doubtful, but any measure was justifiable that held out the faintest prospect for relief. Of course, the ideal operation by analogy would be to open the ventricles.

Dr. Jacob Michaux reported two cases, both ending in recovery.

CASE 1. Aged eighteen months, resulting from or following measles. First, there were dysenteric symptoms, then meningeal, *e. g.*, retraction of the head, distortion of the eyes, convulsions, etc. Calomel was freely administered and the head blistered.

CASE 2. Aged twelve months; had symptoms practically the same, but it was primary. Treatment was the same. He did not use opiates, as he feared the tendency of increased circulation in the brain.

Dr. Landon B. Edwards said Dr. Michaux's cases were streptococcic forms of cerebro-spinal meningitis. There were four cardinal points to be remembered—headache and vomiting, fever and delirium. It was almost impossible to get a succinct history of the case. Tubercular meningitis rarely begins before the second year, and it was not in its nature to recover. At the same time there was a devlopment of tuberculosis in some other part of the body. A diagnostic point was the fever, which was not as high in this as in other forms of meningitis.

M. W. PEYSER, M.D., Secretary.

---

SEMI-CENTENNIAL OF THE AMERICAN MEDICAL ASSOCIATION.

In view of the fact that the next meeting, which is to held in Philadelphia, June 1–4, 1897, will be the semi-centennial gathering, and that it will occur in a great medical center, and near the other great cities of the Eastern coast, the Committee of Arrangements for this meeting have already made provision for the accommodation and entertainment of the delegates by the engagement of the Academy of Music, Horticultural Hall, the South Broad Street Theater, and the large meeting-rooms in the Hotel Walton and Hotel Stenton. As these large buildings are all within a short distance of the great railroad depots in the center of the city, and are all situated within one block on both sides of Broad street, every department of the meeting will be conveniently arranged. At the last meeting of the Association it was voted to devote the first evening of the meeting, Tuesday, June 1st, to dinners of the various sections. The officers of the sections desiring to give such a dinner will please communicate with Dr. G. E. de Schweinitz, Chairman of the Sub-committee on Accommodation, 1401 Locust street, as early as possible in order that dining-rooms may be engaged or other entertainment provided. As it is expected that fully three thousand physicians will be present, the committee suggests that applications for accommodations be made as early as possible. It is hoped that every member of the Association will make a special effort to attend. Further circulars of information will be issued by the committee from time to time.—*Medical News.*

